# Dead or Alive? A Review of Perinatal Factors That Determine Canine Neonatal Viability

**DOI:** 10.3390/ani12111402

**Published:** 2022-05-30

**Authors:** Oliwia Uchańska, Małgorzata Ochota, Maria Eberhardt, Wojciech Niżański

**Affiliations:** Department of Reproduction and Clinic of Farm Animals, Faculty of Veterinary Medicine, Wroclaw University of Environmental and Life Sciences, 50-366 Wroclaw, Poland; o.uchanska@gmail.com (O.U.); maria.eberhardt@upwr.edu.pl (M.E.); wojciech.nizanski@upwr.edu.pl (W.N.)

**Keywords:** dog, pregnancy, parturition, puppy, neonatology

## Abstract

**Simple Summary:**

The article summarizes the current knowledge on factors related to pregnancy, parturition, and newborns that affect the health status of a puppy and determine its chances for survival and development. The detailed information is provided in terms of breed predispositions, objectives of pregnancy monitoring, potential sources of complications, and veterinary advances in care and treatment of perinatal conditions. Successful pregnancy outcomes still pose challenges in veterinary neonatology; thus, publications presenting the current state of knowledge in this field are in demand.

**Abstract:**

The perinatal period has a critical impact on viability of the newborns. The variety of factors that can potentially affect the health of a litter during pregnancy, birth, and the first weeks of life requires proper attention from both the breeder and the veterinarian. The health status of puppies can be influenced by various maternal factors, including breed characteristics, anatomy, quality of nutrition, delivery assistance, neonatal care, and environmental or infectious agents encountered during pregnancy. Regular examinations and pregnancy monitoring are key tools for early detection of signals that can indicate disorders even before clinical signs occur. Early detection significantly increases the chances of puppies’ survival and proper development. The purpose of the review was to summarize and discuss the complex interactions between all elements that, throughout pregnancy and the first days of life, have a tangible impact on the subsequent fate of the offspring. Many of these components continue to pose challenges in veterinary neonatology; thus, publications presenting the current state of knowledge in this field are in demand.

## 1. Introduction

The main goal for dog breeders and their veterinary associates is the successful rearing of puppies. High mortality rates can affect even litters that receive the best care. 

Depending on the cause, it can range from 8 to 20% [1,2,3,4,5]. This paper summarizes the main findings regarding the maternal, pregnancy, and newborn-related components that eventually would determine puppy survival. Analyzing maternal and pregnancy-related factors emphasizes the need for tailored care for each pregnant female. This is particularly true for pedigree bitches, which can be prone to obstetric complications due to the anatomy of their birth canal [6,7,8], as is observed with brachycephalic bitches [9,10]. The combination of female age and body size also affects the potential number of puppies per litter [11,12]. This is an important risk factor, especially for females older than 6 years, for miniature and giant breed females with singleton pregnancies, and giant breed bitches carrying more than 11 fetuses [13,14]. These cases are exposed to primary and secondary uterine inertia, which, as the data in the literature indicate, would pose a direct threat to puppies’ lives [3]. The health status of the dam and the level of care are also of considerable importance. 

Pregnancy is a demanding state that can be significantly affected by the development of any metabolic and hormonal disorders that, if diagnosed too late, can cause serious health complications or even death of both the mother and the litter. Although not all complications encountered by a pregnant or delivering bitch will be fatal, many of them may significantly affect the health of the offspring in those first crucial days and weeks of life. This paper aims to provide and discuss up-to-date knowledge concerning the most important pre- and postnatal factors that may affect the health and survival of puppies during the first weeks of life.

## 2. Maternal and Pregnancy-Related Factors

### 2.1. Anatomy and Breed Predispositions

There is no doubt that of all the factors that affect puppies’ health at birth, maternal factors are the first to play the most important role right from the very beginning of pregnancy. The health status and age of the bitch at the time of conception are most important for the subsequent embryonic and fetal development. Moreover, a full clinical examination should be performed, including a complete blood count and vaccination status, and the general health status should be considered before deciding on breeding a particular animal. During pregnancy, some hematological changes usually occur and should be closely monitored, especially red cell and platelet counts. Gestational anemia and thrombocytopenia are normal findings; however, if excessively low, these may affect the course of pregnancy and lead to clotting problems during the caesarean section.

Undoubtedly, special attention should also be paid to the breed of the dam and the sire breed. Breed identity determines typical anatomical structure and is closely related to the predisposition to perinatal complications [6]. The percentage of these complications increases with the prevalence of certain anatomical characteristics. Among these, the pelvic structure and shape and the skull size are very important for the passage of the fetus through the birth canal during delivery [10]. Serious problems in this regard, due to the disproportionately large size of the fetal head in relation to the size of the birth canal, are encountered in brachycephalic breeds. Bitches of these breeds have a predisposition to perinatal complications almost 11 times higher than in other breeds [10]. This results in an elective cesarean section becoming the norm as natural delivery poses a too high risk for the litter. CT pelvimetry studies have shown that English bulldogs have a significantly smaller pelvis and pelvic canal compared to non-brachycephalic dogs of the same weight. Furthermore, the pelvic conformation is characterized by a significantly shorter pelvis and pelvic canal and a significantly narrower caudal opening of the pelvis [9]. Problems related to pelvic anatomy have also been reported in some medium-sized breeds, such as Scottish terriers and Boston terriers [6,7]. X-ray measurements in bitches diagnosed with birth complications due to fetal-pelvic disproportion showed a smaller pelvis and a dorsal-ventral flattening of the pelvic canal. Whereas, in Boston terrier bitches, the problem was also caused by a combination of the pelvic shape described above and the relatively large head size of mature fetuses. Such characteristics significantly increase predisposition to obstructive dystocia and secondary uterine inertia [6,7,15]. According to a study conducted on a population of ~200,000 bitches registered with Swedish Kennel Club (Stockholm, Sweden) between 1995 and 2002, Scottish terriers were the most susceptible to dystocia resulting in emergency cesarean section [16]. The welfare of breeding dogs has gained increased interest in recent years. Improved understanding that dystocia may pose a greater risk in particular breeds of dogs should contribute to reducing the general popularity of such breeds and help veterinarians and kennel clubs to better focus their resources on strategies to limit breeds with high risk of dystocia [1,17].

In addition to the pelvic bone shape, the structure of the soft tissues in the birth canal can also create a problem. It has been proven that abnormal vaginal anatomy can affect fertility, as it prevents natural mating. The presence of adhesions, septations, or double cervical orifices should be diagnosed before the bitch is intended for breeding [18]. Moreover, if detected, such females should be excluded from breeding, as these defects might be transmitted to the next generation [19]. A study comparing the fertility of bitches undergoing surgical vaginal correction and those without surgery showed that although there was a relatively non-significant effect on reproductive performance, pregnancy rates were substantially lower in the group of bitches with severe abnormalities. Furthermore, the same group of bitches showed a markedly greater predisposition to dystocia and thus cesarean section than the group of bitches with mild abnormalities [8]. 

### 2.2. Inbreeding

When analyzing the causes that could affect the litter size, inbreeding should also be considered. According to research, a high inbreeding rate can result in impaired fertility and therefore a reduction in the number of pups in the litters that are born [20]. In pedigree dogs, breeding decisions on mating closely related individuals are motivated by the need to obtain the desired phenotype or behavioral traits, leading to genetic bottlenecks. Targeted selection in pedigree dogs for traits of morphology and performance has greatly decreased fertility rates, increased reproductive problems, and significantly contributed to dystocia due to anatomical disproportions in some breeds [10]. The most notable among these are brachycephalic breeds, in which reproduction without assisted mating and a planned caesarean section is today almost not feasible [21]. Focusing on breeding for desired appearance alone raises a lot of public concern today, which hopefully will lead to better awareness and more responsible dog breeding in future. 

The availability of genetic tests dedicated to particular breeds is also worth mentioning. Today, the problem of selecting pairs of animals for breeding rests mainly on the responsible approach of the owner, and any poorly considered actions can lead to the perpetuation of traits detrimental to the health of dogs and the narrowing of their genetic pool [22]. The number of breed-specific conditions increased at more than 100% between year 2013 and 2020 [23]. The range of dedicated tests for their identification is also growing steadily [23]. Among the 10 breeds for which test availability is the greatest, are Labrador Retriever, Beagle, Australian Shepherd, German Shepherd, Standard and Miniature Poodle, Golden Retriever, Collie, Pembroke Welsh Corgi, and Dachshund [23]. Detailed information on the range of genetic tests for detecting inherited diseases can be accessed online at: The WSAVA-PennGen DNA Testing Database, which is part of “A project of the WSAVA Hereditary Disease Committee” [24]. 

### 2.3. The Impact of Litter Size

When considering the influence of maternal factors, one cannot forget to mention their effect on the litter size. The litter size directly affects the safety of puppies during birth and depends on the mother’s age, breed, and the breeding method [11,12]. The larger the breed, the more pups a litter would potentially contain (up to 11–12 pups), whereas in small and miniature breeds on average, up to 3–5 pups are more common [11,13,14]. 

Large females have a larger uterine capacity and are able to accommodate more fetuses; hence, their litters are naturally more numerous than those of smaller females. In larger breeds, the small size of the litter is especially important because a small number of pups may not give enough strong signals to induce labor [25]. Furthermore, multiple litters of 12 or more pups can cause primary uterine inertia by overstretching the myometrium, leading to weak uterine contractions, or secondary uterine inertia, where the uterine myometrium is exhausted by prolonged labor [6,11,13].

Regardless of the litter size, the last pup is at the highest risk of stillbirth [6]. Some authors pointed out that high perinatal mortality most often affected the first pregnancy of a given dam; in addition, the risk of early neonatal mortality nearly doubled in litters with a concurrent incidence of stillborn pups during birth [13]. 

Large litters can also be associated with risks to maternal health. Among other things, it can be one of the factors contributing to the bitch’s disease during pregnancy and it can increase the risk of dystocia. Gestational toxemia, for example, occurs most often secondarily to a negative energy balance associated with inadequate nutrition and largely with the presence of a large litter of puppies [26,27].

The importance of maternal age played a role in very young (1 year and under) and older (6 years and older) bitches, that tend to have fewer pups (one or two) than bitches aged 2 to 5 years [13,14]. It is worth noting here that primiparous bitches older than 6 years are predisposed to single-puppy pregnancies, uterine disorders, and prolonged labor [3,13]. Hence, such factors would significantly increase the risk of dystocia, and it is highly inadvisable to breed bitches over 6 years of age [13].

The mating method can also be a limiting factor in terms of the number of offspring. The most commonly used method is natural mating or artificial insemination (AI). AI can be intravaginal, intracervical, or surgical, using fresh, chilled, or frozen semen [28,29]. The highest efficiency in obtaining numerous litters was reported through natural mating rather than with the artificial insemination method [11]. However, natural mating has become less and less popular among dog breeders, mainly due to the need to travel long distances for a chosen male. This makes breeders much more eager to choose the artificial insemination methods and shipped semen [29]. The use of the artificial insemination method also provides highly satisfactory results in terms of litter size, especially when using fresh semen rather than chilled or frozen-thawed [12,30].

### 2.4. Maternal Health Condition

The course of a pregnancy fundamentally determines the health of a newborn during its first weeks of life. The number of factors that may affect the mother’s condition during this period is innumerable. They can be divided into those related to the bitch, such as current health state or ability to maintain hormonal balance, and those that are external: mainly the diet and the quality of pregnancy monitoring. During the pregnancy, several disease entities can also develop such as gestational toxemia, diabetes, and eclampsia. The pregnancy can also be affected by corpus luteum insufficiency, which is a serious disorder at the ovarian level [26,31]. Awareness of the potential risks to the mother and the level of care provided if complications arise are vital and can significantly impact the future of any litter.

#### 2.4.1. Pregnancy Toxemia and Diabetes Mellitus

Canine pregnancy toxemia can pose a serious life threat to both the bitch and her fetuses. The disorder, which is mainly seen in late pregnancy, is characterized by hypoglycemia, ketosis, ketonuria, and liver lipidosis, presenting with weakness, which can later progress to seizures, collapse, and even death [32]. Bitches with multiple litters are more at risk of developing toxemia, especially if they show signs of anorexia during the last two weeks of pregnancy [18]. The diagnosis is based on clinical signs and the presence of hypoglycemia combined with high concentrations of ketone bodies in blood and urine, and treatment involves glucose supplementation [15,32,33].

Symptoms similar to gestational toxemia may also be observed during gestational diabetes, which is often developed without the typical clinical signs evident. A syndrome resembling gestational diabetes in humans may occur in older female dogs during pregnancy. Insulin resistance is a normal feature of pregnancy [33]. Two factors promoting insulin resistance, progesterone and growth hormone, are present in similar concentrations in pregnant and non-pregnant bitches in diestrus [34]. Progesterone stimulates the secretion of growth hormones from the canine mammary gland, and both hormones cause insulin resistance and carbohydrate intolerance in the dog [35]. However, pregnant bitches are more insulin-resistant than non-pregnant diestrus bitches [36]. The diagnosis of gestational diabetes mellitus (GDM) is based on history and clinical findings and is confirmed by documenting the persistent hyperglycemia with glucosuria. In humans, GDM is associated with excessive fetal growth (macrosomia), which contributes to dystocia and birth trauma, and, as a result, increased maternal, fetal, and neonatal morbidity and mortality [37,38]. In humans, the prevalence of congenital malformations is higher than in the general population if GDM occurs early in pregnancy, but not in mid-pregnancy or later [39]. In bitches diagnosed with gestational diabetes mellitus, fetal viability should be assessed regularly with ultrasound; knowing whether the pups are large enough to be at risk of dystocia or are no longer alive would guide treatment decisions, such as the schedule of the cesarean section. Such puppies would be at increased risk of premature delivery if CS was decided too early, while if delayed too long, it would lead to fatal distress and metabolic disorders. Published reports of managing canine GDM are rare [35,36,40]. Similar to humans, in case of GDM neonatal hypoglycemia, hyperbilirubinemia, hypocalcemia, and poor suckling can occur more frequently in puppies, and proper management involves intensive fluid and insulin therapy to correct water, electrolyte, acid-base, and glucose derangements [33,40].

#### 2.4.2. Hypoluteoidism

Hypoluteoidism is described as insufficient production and secretion of progesterone by the corpus luteum [41]. Necessary to maintain pregnancy, proper corpus luteum function and progesterone production, if defective, can determine the fate of any pregnancy at its very early stage. A serum progesterone level of at least 2 ng/mL is considered by many authors to be the key to maintaining a canine pregnancy [34,42]. A decrease in progesterone concentration below 2 ng/mL in a pregnant bitch has been shown to lead to abortion [34,42]. Interestingly, some authors reported that miscarriage can occur even when the progesterone level drops below 10 ng/mL [43]. Since serum progesterone measurements can be performed using a variety of methods and devices on the market, with RIA being considered the reference assay, the exact progesterone values may vary between published studies and reports [44]. In all cases, the interpretation of the results should be approached with caution and the device and method used should always be considered when analyzing the progesterone concentration.

Distinguishing between corpus luteum insufficiency as the primary cause of fetal death, rather than its consequence, is certainly worth attention, especially in bitches who lose litters in the second half of pregnancy despite the exclusion of other causes. The reasons and pathogenesis of corpus luteum insufficiency, despite many studies, are still incompletely investigated and diagnosis often poses a problem because many factors, such as maternal health status, fetal death, infectious agents, trauma, or poor nutrition, may contribute to the primary decrease in progesterone level [34,45,46]. Some studies confirmed the association of corpus luteum insufficiency with the presence of IgE antibodies against endogenous progesterone in bitches [47]. These authors suggested a genetic link to the occurrence of hypoluteoidism in specific breeds, such as German Shepherds. 

The diagnosis of primary corpus luteum insufficiency as a cause of pregnancy loss is complex. It requires confirmation of a gradual decrease in progesterone levels while assessing fetal viability. The possibility that the decrease in serum progesterone levels occurred due to fetal distress or abortion must be excluded. The function of the corpus luteum can be influenced by many endo- and exogenous factors that control its lifespan, and among the most important substances with luteolytic effects are PGF2a and antigestagens such as aglepristone [46,48].

If the diagnosis is confirmed, exogenous progestin supplementation therapy is recommended, of which medroxyprogesterone acetate (MPA), alternogest, or progesterone in oil are the most commonly used in dogs [34,49]. The administration of progestins in pregnant bitches may be associated with side effects such as the development of pyometra, septicemia, or placentitis, and the risk of prolonged pregnancy-causing dystocia [34,42,50]. Furthermore, progestogens, which have androgenic effects, may lead to masculinization of female fetuses [42]. 

Recently, the first documented case of mammary gland fibroadenoma in dogs, so far reported only in cats, has also been described. An Istrian Shorthaired Hound bitch was diagnosed with primary corpus luteum insufficiency in three consecutive pregnancies [51]. Based on the diagnosis established during the first pregnancy, in the second one with repeated suspicion of corpus luteum insufficiency, the bitch was treated with 1.65 mg/kg of progesterone in oil intramuscularly (PROGEST-E^®^, Fort Dodge Animal Health S.p.A., Bologna, Italy) daily, from day 19 to day 22, and every 48 h from day 23 to day 58. During the third pregnancy, based on previous experience, the bitch also received 0.075 mg/kg alternogest (Regumate^®^, Intervet Italia S.r.l., Peschiera Borromeo, MI, Italy) from day 8 and then every 24 h until day 52 of pregnancy. The dose was reduced to 0.058 mg/kg PO from day 53 to day 57 [51]. Both times, an increase in the size of the mammary glands was observed. One of the pregnancies required a cesarean section due to fetal macrosomia. Whereas, females born in the other pregnancy, according to the owner’s report, showed no signs of estrus cycle during the first 3 years of life; moreover, in one female, clitoral hypertrophy and a blindly ending vagina were diagnosed. Such abnormalities may be related to progestin treatment in the embryogenesis stage, as they affect the development of the genital tract. If progestins are used as a supplemental treatment for hypoluteoidism, administration should not begin before days 30–35 to avoid genital abnormalities [49,52]. Since hypoluteoidism is an ovarian dysfunction and its treatment with progestins might lead to severe unwarranted effects, it is recommended to exclude affected bitches from breeding.

#### 2.4.3. Hypothyroidism

An equally interesting topic is the relation between pregnancy and hypothyroidism. In general, hypothyroidism is a common endocrine disease in dogs. Many previous studies have demonstrated a heritable tendency in dogs of certain breeds, such as Toy Fox Terriers, Giant Schnauzers, Boxers, or Scottish Deerhounds [53]. Bitches with untreated hypothyroidism often exhibit a significant reduction in fertility that prevents natural pregnancy, as well as variable interestrus intervals, abortions, and stillbirths [54]. Studies have shown that they are also at risk of developing dystocia as a result of a prolonged duration of labor contractions and a decrease in their intensity. Puppies have been reported to have a higher incidence of low birth weight, low viability, and overall increased litter mortality [54]. The situation changed when levothyroxine supplementation was started, which was shown to reverse the negative effect of hormone deprivation on neonates’ birth weight and high mortality [55]. The question of changes that occur in thyroid hormone concentrations during the course of pregnancy in bitches certainly requires further study. Some data indicated insignificant fluctuations that do not require adaptive hormonal supplementation during pregnancy [56,57]. In contrast, others suggested that significant changes may occur, especially during the second half of pregnancy, when the rate of fetal development increases, and therefore the rate of maternal metabolism should follow to meet the energy demands [42,58]. The same is true in human pregnancy, where adequate supplementation during pregnancy is necessary for its maintenance and must be gradually increased, as well as the fetal development and metabolic rates [59]. In bitches experiencing miscarriage, a significant decrease in thyroid hormones and a correlating decrease in progesterone levels were observed even one week before the first clinical symptoms [42]. The problem of levothyroxine supplementation recommendations and the need to increase the dose with the progression of pregnancy certainly require further research and attention; this is still a matter of debate according to different study results [56,57,58], because decisions made during treatment can have a significant impact on the outcome of the bitch’s pregnancy and therefore the health status of the whole litter. 

#### 2.4.4. Maternal Microbiota and Its Effect on Puppy Survival

The development of the newborn microbiota is a gradual process, influenced by many factors. The environment, the health status of the mother and, above all, the intake of colostrum, are essential in the first hours of life [60].

The colostrum in mammals is the first food of crucial importance for the health of the offspring in the early neonatal period. In canine species, only 5–10% of antibodies can cross the placenta, so newborns are almost completely immune-deficient [61]. The timing and amount of colostrum intake immediately after birth are important for their survival and future health. The intestinal barrier is only permeable to IgG from the gastrointestinal tract during the first 12–16 h of life, so it is important to feed newborns with colostrum as soon as possible shortly after birth [61]. Although the exact mechanisms that determine the formation of the colostrum microbiota are not fully understood, studies seem to support the enteromammary hypothesis that the colostrum microbiota is shaped by gut bacteria present in the dam [61].

The relationship between colostrum microflora and intestinal microflora of neonates is confirmed by studies in which the composition of meconium collected from neonates was found to be similar to that of colostrum collected from dams [61]. Adequate microflora composition determines colonization of neonatal intestines, and this directly translates into their chances of survival, adaptation to the ectopic environment, and development of health status [60].

Studies on supplementation of pregnant bitches with prebiotics containing, among others, *E. faecium* and *L. acidophilus* proved their influence on producing better quality colostrum, which improved the immunity of puppies. Furthermore, the maternal microbiota, positively altered by supplementation, was transferred to newborns, making the offspring more resistant to gastroenteritis [62].

The type of parturition can also be a factor that affects the composition of the microflora of the colostrum [61]. Whether it is vaginal delivery, emergency cesarean section, or elective cesarean section, the colostrum samples contained different microorganisms. The colostrum of the bitches with vaginal delivery and elective cesarean section was characterized by a similar composition, unlike the colostrum collected from the bitches undergoing emergency cesarean section [61]. It should be noted that the differences may result from the fact that bitches in the latter group usually experience higher levels of stress as well as some degree of exhaustion. 

Recent findings concerning the placental microbiome in healthy pregnant dams also indicate the possibility of early intrauterine bacterial colonization of the fetuses [63]. The bacterial composition of the neonatal meconium resembled that of the maternal vagina in the pups born by vaginal delivery. The ones born via CS had microflora similar to the oral and vaginal microbiota of the mother [63].

It should also be noted that the presence of the gut microbiota can contribute to better weight gain in puppies, which is essential for their future development and health status in adulthood [61,63]. Puppies in which bacteria could not be cultured from meconium had a slower growth rate compared to puppies with some bacteria present in their meconium [63].

It is also extremely important for the health of the offspring to maintain the balanced composition of their intestinal microbiota. The underdeveloped immune system can be easily affected by the gut microflora imbalance, which may lead to the growth and multiplication of undesirable pathogens such as Proteobacteria and Pasteurellaceae, and may progress towards serious disorders, mainly Fading Puppy Syndrome (FPS), which can often cause fatal results [64]. The evaluation and culture of the intestinal microflora composition and the fecal samples of puppies can therefore become a helpful tool in the diagnosis of neonatal problems.

#### 2.4.5. Challenges in Pregnancy Nutrition

Proper nutrition has an important impact on the health of the mother and her offspring during pregnancy and the subsequent lactation. The connection between reproduction and nutrition is undeniable and awareness of key aspects of bitch nutrition during pregnancy, perinatal period, and lactation is a prerequisite for success in healthy breeding. Facing differences in reproductive animals’ nutritional needs, Association of American Feed Control Officials (AAFCO) has published separate nutrient profiles with different feeding trial protocols for maintenance, growth, and gestation-lactation periods [65]. Energy requirements gradually increase in the bitches from day 40 of gestation, on average by nearly 10% for each additional week, to about 1.25–1.5 times those of normal maintenance requirements. The amount of food provided should be properly recalculated to prevent excessive weight gain [66]. Many owners believe that large amounts of energy are required from the very beginning of the pregnancy. They start to overfeed pregnant bitches, which can result in increased fat deposition [66]. Unfortunately, obesity is one of the factors that strongly predispose to dystocia, which most likely decreases uterine contractions during labor due to excessive fat deposition in muscle tissue [6,67]. It is also worth noting the practice of dividing the daily dose into several smaller meals. It prevents the stomach from overloading with too much food, especially in late pregnancy, when a large part of the abdominal cavity is filled with the gravid uterus [66].

Just as important as quantity is the quality and type of food provided. A study conducted among a group of breeders in Canada about their feeding practices has shown that a substantial percentage of breeders were unable to determine whether the commercial food they were feeding met the AAFCO recommended standards. In addition, nearly 16.9% of breeders participating in that survey, who fed commercial diets, reported offering diets to pregnant and lactating bitches that are not intended for the gestation-lactation period. Moreover, almost 9% of breeders weaned puppies onto a diet not designed for the growing puppies [68]. An alternative to commercial diets available on the market is the bones and raw food diet (BARF), prepared by owners or breeders at home, sometimes with specific additives [69]. Its nutritional value for pregnant and lactating bitches and growing pups is difficult to assess and very likely BARF diet would not meet the demands of breeding animals [68,69]. This type of diet may also increase the risk of infection with several pathogens and parasites responsible for reproductive disorders, including *Salmonella* spp., *Campylobacter* spp., *Neorickettsia* spp., Shiga toxin and hemorrhagic *Escherichia coli*, *Toxoplasma* sp., and *Neospora* sp. [68].

When selecting foods for pregnant and lactating bitches, attention should also be paid to the content of the particular ingredients and the use of various additives (e.g., mineral supplements, dairy products); breed-related demands should also be considered here, especially regarding calcium content [66,70]. Some breeders believe that calcium addition can ensure adequate lactation and healthy development of puppies [66]. However, a lack of consideration in calcium supplementation may cause more harm than benefit. Calcium homeostasis is tightly regulated by parathyroid hormone (PTH) and calcitonin [71]. During pregnancy, a physiologic decrease in calcium serum levels leads to a series of reactions designed to elevate it again. Under the action of parathyroid hormone (PTH), an increased release of calcium from bones and enhanced absorption from the gastrointestinal tract takes place [70,71]. When a bitch receives extra calcium with food, the serum calcium concentration remains sufficiently high, and there is no need to activate the mechanisms of calcium release from bones and increase the intestinal absorption, which downregulates the PTH activity. Then, with the onset of lactation, a bitch cannot precisely respond to the high calcium demand and the risk of developing eclampsia increases [66]. Eclampsia most often occurs in small and miniature breeds, usually after birth, but sometimes might also be observed during pregnancy [15]. Clinical signs are initially nonspecific, but can progress to muscle tremors, ataxia, elevated rectal temperature above 39.7 °C, tetany, and in severe cases, even death [70]. Treatment is based primarily on intravenous calcium administration. If possible, the litter should be weaned permanently and hand-reared to prevent the risk of hypocalcemia relapse. 

Another nutrient that has also attracted interest is folic acid, recommended for pregnant women. Experiments conducted on mice as experimental animal models also demonstrated that folic acid is necessary for normal embryonic-fetal development [72,73]. Some studies have shown its important role in the prevention of cleft lip and/or palate in brachycephalic puppies [74,75,76]. However, no similar relationship was proven in a study conducted on bitches of breeds considered as non-predisposed (Labrador, Golden Retriever, and Labrador/Golden crosses) to the cleft palate problem [77]. If considering supplementation in dogs, studies suggest one should start folic acid when a bitch is intended for breeding/enters the estrus phase, since the medullary tube closes during the first part of gestation to reduce the risk of developing cleft lip or palate [66]. 

The crucial importance of a proper diet for the health of the pregnant bitch and her offspring remains undisputed. In addition to portioning daily food intake and administering it in appropriate amounts to avoid excessive weight gain, the trend of administering certain supplements deserves attention, especially calcium and folic acid supplementation. A rational approach to the use of nutritional supplements and the application of sound feeding practices are key aspects in providing adequate care to the pregnant bitch.

#### 2.4.6. Pregnancy Monitoring

In routine care of pregnant females, periodic examinations are recommended to evaluate the pregnancy development. US pregnancy scanning, if performed by an experienced person, would allow for early detection of abnormalities. Defective trophoblast invasion is believed to be the basis for abnormal blood flow in the uterine artery, which in turn leads to uteroplacental insufficiency [78]. Alarming changes include reduced diameter and abnormalities in the contour of the gestational vesicle, lack of viability, increased placental thickness, increased fluid echogenicity, and increases in RI (resistivity index) and PI (pulsatility index) of uteroplacental arteries of conceptuses [78]. The appearance of changes correlates with the onset of embryo resorption around weeks 2 and 4 of gestation [78]. The umbilical artery and the fetal renal artery were also useful in evaluating the quality of fetal-maternal flow; its abnormal parameters suggest gestational pathology [79]. Differences were also reported in the blood flow of the uterine and umbilical arteries in small, medium, and large breeds of dogs during the second half of pregnancy. Conversely, during this same period, FHR did not vary between small, medium, and large breeds [80]. This indicates that physiological variations should also be considered when a gestational ultrasound is interpreted in different breeds of dogs. Doppler ultrasound also has a prognostic value in assessing the effect of the morphology of the ductus venosus (DV) waveform on canine neonatal mortality. One study showed that litters in which three-phase waves of DV (tDVw) were recorded had a higher (almost 21 times more) chance of neonatal mortality (one or more dead pups per litter) than those with only two-phase waves (dDVw) [81]. DV postnatal patency could also lead to a congenital portosystemic shunt (CPSS). Non-invasive color flow mapping (CFM), which is used routinely to diagnose CPSS in adult dogs, has been shown to be equally useful to confirm or exclude DV closure within the first ten days after delivery in canine neonates. This might become useful for early screening tests evaluating the DV patency, especially in puppies of breeds predisposed to congenital portosystemic shunts [82]. Changes observed during ultrasound examination may indicate the development of pregnancy pathology long before the first visible symptoms appear [83].

In addition to ultrasonography, electrocardiography can sometimes be useful to detect pathologies in specific electrocardiographic parameters, including QRS waveforms, maternal heart rate (MHR), or fetal heart rate (FHR) [84]. The correct MHR should be around 70–120 bpm, while the fetal heart rate is between 180 and 220 bpm [85]. Any factor causing fetal distress is most often manifested by fetal bradycardia and, therefore, changes in FHR. A more complete picture of the disorder can be obtained by analyzing the FHR/MHR ratio, which more accurately reflects fetal health in relation to maternal health than single FHR values [85]. This is especially the case when combined with Doppler examination, which enables one to visualize and compare the blood flow of the uteroplacental and umbilical arteries in normal and abnormal conceptus [78]. Accurate understanding of the changes that occur can contribute to early diagnosis for identification and, if possible, exclusion of the problem to ensure the safety of the developing offspring.

## 3. Type of Delivery, Perinatal Complications, and Proper Management of the Bitch and Her Offspring

### 3.1. Normal Parturition

The pregnancy in the bitch is expected to last between 57 and 72 days from the day of mating [40,86]. Estimating the exact date of parturition in dogs is sometimes difficult. The timing of ovulation is usually delayed in relation to both the LH surge and the liberation of the immature ovum, which needs to mature further to be competent for fertilization in the oviduct [87]. It is also necessary to consider the 24–48 h period in which the ovum can be fertilized and the survival of the sperm in the reproductive tract, depending on the date of mating or the type of insemination. 

Serum P4 concentration is considered an essential parameter for the detection of the LH peak, ovulation, and parturition date. Serum P4 concentration begins to increase before the onset of the luteal phase, and the first day when P4 is >1.5 ng/mL is considered to indicate the peak of LH [88]. On the day of ovulation, which occurs almost 48–60 h after the peak of LH, the length of pregnancy is reported to be 63 ± 1 days (62–64 days) [40,88]. The accuracy of parturition timing using prebreeding P4 concentration is described to be 67, 90, and 100% within 65 ± 1, ±2, and ±3 days, respectively [88]. Instead, vaginal cytology for continuous monitoring of the reproductive cycle could be helpful in determining the appropriate time to start serum P4 monitoring [86]. Vaginal cytology can also be used to determine the first day of diestrus (D1), after which the pregnancy should last 57 ± 3 days (54–60 days) [86]. The determination of D1 does not present difficulties because the image in this phase of the cycle clearly differs from that in estrus by the decrease in the percentage of cornified cells from 80–100% to at least 20%, and the appearance of parabasal cells and neutrophils. These changes occur in less than 24–48 h [86].

A normal parturition in a dam is divided into three phases. In phase one, the progesterone block is lifted with the onset of uterine contractions and cervical dilation. The duration varies between 6 and 24 h, possibly up to 36 in a primiparous or very nervous bitch [40]. During the second phase of labor, uterine contractions are combined with abdominal wall contractions, and the fetuses are expelled sequentially from the uterus. Although there may be a longer interval between the first and subsequent pups, births usually occur every 15 to 120 min, depending on the size and breed of the mother and the number of puppies. The third phase involves placental expulsion and occurs immediately or within 15 min after the birth of each pup [40]. However, several placentas may be delivered at once [89].

### 3.2. First Aid Delivery

Labor, for a variety of reasons, does not always proceed as expected and veterinary assistance may be required when complications occur. A fast and accurate diagnosis of dystocia is essential to increase the chances of survival of a newborn. The most important predisposing factors to dystocia are those previously discussed, including maternal age, size, breed, and the number of puppies [6]. Difficult labor can be approached with conservative methods or surgical intervention. Conservative methods involve the use of pharmacological substances and/or manual attempts to correct the fetal position in the birth canal [5]. When trying to pharmacologically resolve difficult labor, oxytocin is administered most frequently [2]. Calcium gluconate and glucose are also recommended [89]. If there is no response to drug therapy or obstetric maneuvers aimed at correcting dystocia, and fetuses are confirmed to be alive, the dam should immediately be submitted for an emergency cesarean section [5]. When considering the potential impact of the cesarean section on the health of puppies, two key things should be remembered. One is related to whether the cesarean section is performed as an emergency or a scheduled procedure, and the other is the type of anesthetic protocol used during the intervention. 

### 3.3. Emergency Cesarean Section

The emergency cesarean section is a procedure associated with a high risk to the life of both the bitch and the puppies. Mortality rates can be as high as 20% of puppies and 1% of bitches, especially in small and predisposed breeds, including predominantly brachycephalic breeds like French Bulldog, Boston Terrier, Chihuahua, or Pugs [1,2,3]. 

The most common maternal causes include difficult fetal passage (narrow birth canal, presence of undetected earlier congenital vaginal defects), disturbed labor (inertia, spasms), prolonged pregnancy, uterine disturbances (torsion, malformations), and poor psychogenic status (restlessness, abnormal or aggressive behavior) [1,3]. Fetal factors include puppy size, especially oversized ones, fetal malpresentation, and the total number of the puppies [3]. Single pup pregnancies or abnormally high numbers of pups should always be treated with special care because they are serious predisposing factors to delivery complications.

The longer it takes to deliver a puppy and the later the surgery is started, the less chance of survival a puppy has. Severe fetal distress translates into heart rate per minute; a drop below 180 bpm is an immediate indication for the surgical intervention, whereas below 120 is a very poor prognosis for the puppy’s survival [21,90]. The published data directly indicate that puppies born via emergency CS are more than 7.3 times less likely to survive than those born via elective CS [21,91]. The most common cause of the pup death is hypoxia due to prolonged labor and placental separation, often associated with large numbers of fetuses, malpresentation, and wedging in the birth tract [3,92]. 

### 3.4. Planned Cesarean Section

One of the ways to prevent puppy loss due to dystocia is to schedule a cesarean section. Specific indications for an elective CS are breed predisposition, maternal age, and litter size. Females with previous birth complications and those of high breeding value should also be considered. It has been proven that in some situations, scheduling a cesarean section is the safest method of pregnancy termination both for the dam and her litter [91]. However, performing a cesarean section without clear indications at the owner’s own request is the subject of much controversy and is considered unethical by some members of the veterinary community. In the case of scheduled operation and for the safe surgical outcome, the appropriate anesthetic protocol should be used, which considers both the dam and the fetuses in the uterus. 

In each of the types of delivery, the puppy viability assessment should be performed and adequate treatment instituted if abnormalities are detected [5,93]. Timing is very important for puppy survival, further development, and overall health status. 

### 3.5. Neonatal Assessment

Neonatal viability can be evaluated using a modified APGAR score proposed by Veronesi [94]. Five parameters are assessed, including mucus color, heart rate, reflex irritability, motility, and vocalization. Points (0–2) are given for every parameter depending on the condition of the newborn [94]. A heart rate above 220 bpm (beats per minute) gets 2 points, between 220 and 180 gets 1 point, and below 180 gets 0 points. Respiratory effort is assessed when the newborn is crying (>15 respiratory rate (rr)), moderately crying (between 6 and 15 rr). Reflex irritability can be vigorous, a grimace alone can be visible, or there may be no response. When assessing motility, it is possible to observe active motion, some reflections, or flaccid movements. The last parameter, mucus color, can be assessed as dark pink, pale, or cyanotic. The total amount determines the final APGAR score, which identifies the degree of a newborn’s distress: 7 to 10 points mean no distress; 4 to 6, moderate distress; and 0 to 3, severe distress. 

For brachycephalic breeds born by cesarean section, an exclusive scale has been designed for the evaluation of brachycephalic newborns [95]. The scale was specially modified after the Veronesi et al. scoring system and adapted to their characteristics due to the lower degree of vitality after birth often observed in newborns of these breeds. A heart rate above 180 bpm gets 2 points, between 120 and 180 gets 1 point, and below 120 gets 0 points [95]. Other parameters including respiratory effort, reflex irritability, motility and mucus color are scored the same as in the APGAR scale proposed by Veronesi et al. [94]. 

Neonatal viability reflexes (NVR) can also be assessed. The purpose of this scale is to evaluate a newborn’s postnatal depression based on active searching for the mammary gland and the strength of the sucking, measured as weak, moderate, or normal [5]. 

These reflexes are essential in the early postnatal period to ensure newborn feeding and survival. The suckling reflex can be assessed by inserting the gloved tip of the smallest digit into the mouth of the neonate and checking the suckling force; it can be described as strong (5 suckles/min), weak (>3 suckles/min) or absent. The rooting reflex can be assessed by approaching the nose of the neonate with the forefinger and thumb shaped into a circle and checking whether the neonate inserts its nose into this circle; immediate, slow or absent muzzle fitting inside the circle can be observed. The righting reflex of the neonate can be evaluated by placing it on its back on a soft surface and verifying whether it is able to return to the sternal recumbence. Those reflexes are also scored from 0 to 2 points, and the interpretation is as follows: 0–2 points: weak viability; 3–4 points: moderate viability; 5–6 means normal viability. Weak reflexes are often the primary sign of hypoxia in puppies [5]. 

The better the viability of the newborn, the higher score it receives on each scale mentioned above, and therefore the prognosis of its short-term survival would be better. The transient decline in vital functions immediately after birth is often observed even in eutocic pups [5]. Healthy newborns quickly regain their vitality; however, due to the special vulnerability in the first few hours of life, each should receive special attention and care. The critical and weak puppies (0–6 on the APGAR score) need even more attention and care, as they are much more likely to have a fatal outcome within the first 2 h of life [5].

To increase the chances for survival of weak newborns, prompt and efficient resuscitation is crucial. The protocol proposed by Traas et al. adopted the following sequence of actions according to their importance: warmth, airway, breathing, circulation, and drug administration [96]. In each case, the treatment should consist of clearing airways, drying of a puppy, respiratory stimulation (cardiopulmonary resuscitation), oxygenation, ventilation by a mask or endotracheal tube and, when necessary, fluid administration [5,96]. Intravenous administration of drugs such as doxapram, aminophylline, or epinephrine could also be considered [96]. Naloxone can be given to reverse the anesthetic effect of opioids administered to the dam prior to the cesarean section. In distressed newborns at risk of sepsis due to hypoxia-induced bacterial translocation, the administration of safe antibiotics (these include cephalosporins, penicillins, clavulanic acid, macrolides, trimethoprim-sulfonamide, and amikacin) should be considered [18,96].

Blood glucose measurements are also helpful in assessing the risk in frail puppies. Hypoglycemia (<40 mg/dL) within the first 8 h of life was associated with high mortality in newborns during the first 24 h of life. At 24 h of life, the level of 92 mg/dL or below was associated with a higher risk of mortality during the entire neonatal period (1–21 days) [4]. 

A second useful parameter for predicting neonatal mortality within the first 48 h of life is the lactate level. Its concentration in the umbilical cord blood reflects the presence of acidosis in a neonate, with high levels noted in distressed pups and low levels in healthy, vigorous ones [97]. Both parameters combined with the neonatal APGAR score can be a useful tool for the early identification of weak newborns that require special care, thus reducing neonatal mortality. 

Awareness must also be given to the fact that each evaluation should always be objectively performed by experienced practitioners. Low scores do not always determine a negative outcome for a puppy; however, high neonatal mortality is more frequently correlated with it [90]. When performing a viability evaluation, each newborn should also be checked for the presence of birth defects such as cleft lip/palate, presence of a hernia, or atresia ani.

### 3.6. Postoperative Pain Management

An important aspect that should not be overlooked is proper pain management for the dam after a cesarean section [98]. In dogs, as with all mammalian species, the mother-newborn bond is crucial for the suitable development of maternal behavior that guarantees offspring survival. In dogs, both natural birth and assisted delivery might be a stressful and painful experience, which could affect the way a bitch cares for her litter [99]. A female in pain may present a weaker maternal instinct and refuse to care for or feed her puppies. This, in turn, puts them at risk of developing hypoglycemia and hypothermia, both very dangerous in newborns [100,101]. However, when administering analgesics to the bitch, one must be aware of their ability to pass through the mammary gland into milk. The most commonly used analgesics belong to nonsteroidal anti-inflammatory drugs (NSAID) or opioids [101]. Due to the risk of damaging the immature kidneys and liver of the newborns in the case of using NSAID, or being more sensitive to the sedative and respiratory depressant effects of opioids, especially in pups under three weeks of age, not many drugs are approved for use in pregnant and lactating bitches, significantly affecting their post-surgical recovery and welfare [101]. Studies have shown that cimicoxib and carprofen exhibit low penetration through the mammary gland barrier [101,102]. Treatment of lactating bitches for a short period of time seems to be safe for the offspring; however, carprofen should be used with caution in bitches with diagnosed mastitis, in which case the drug can penetrate the milk in higher amounts [101].

All of the previously mentioned issues are only a fraction of the knowledge necessary to ensure a safe pregnancy outcome and the health of the litter. In the case of emerging complications, the correct diagnosis and prompt treatment are essential. Providing proper care for both the mother and the offspring would significantly reduce the risk of neonatal mortality.

## 4. Newborn’s Related Factors

Birth is the moment of fetal-to-neonatal transition which requires very complex adaptive changes which need to occur in a short period of time. Adaptation failures will result in newborn compromise and homeostasis disturbances, and will determine the success of its rearing. During the transition period, the neonatal condition is self-occurring or caused by factors beyond the newborn’s control, but also the health status and behavior of the mother, as well as improper care and environmental conditions, may play a role in the newborn’s welfare and development [92,103]. 

### 4.1. Fetal Congenital Malformations

An important issue that can affect intrauterine life or the health status of the newborns is the presence of congenital malformations. The canine congenital malformations are structural or functional abnormalities present at birth that may interfere with the viability of newborns, thus contributing to neonatal mortality [89,104]. They can be caused by genetic factors, inherited and/or breed-related, or exposure during pregnancy to teratogenic agents (toxins, chemicals, irradiation, or excessive supply of vitamins A and D) [104]. Studies that examine the frequency of malformations have shown that the most common ones include: cleft palate (2.8%), hydrocephalus (1.5%), anasarca (0.7%), cleft lip (0.6%), polydactyly (0.5%), segmental intestinal aplasia (0.4%) or atresia ani (0.4%), and others [104]. The pups with diagnosed malformations in the above study were 6.7% of 803 animals examined. The mortality rate caused by these abnormalities among the pups during the first two days of life was recorded at 61.4%, while from day 3 to day 30 it was 38.6%. In the vast majority of cases, single malformations were present [104]; much less frequently, simultaneous occurrence of two or more in one fetus was observed [105]. The risk of malformations is much more common in pedigree dogs, and the brachycephalic dogs are among the most predisposed breeds [2,21]. The accumulation of genetic defects over the years due to inbreeding or selective breeding leads to reduced genetic diversity and increased predisposition of these animals to present anatomical anomalies [10]. However, not all anomalies are associated with an increased risk of mortality. For example, puppies born with additional fingers (polydactyly) are unlikely to suffer from other health problems. The vast majority of congenital malformations do, however, have a marked effect on longevity; in the case of anasarca, hydrocephalus, cleft palate, atrophied intestine fragments, or atresia ani, the life span will be substantially affected. Puppies with a cleft palate often develop aspiration pneumonia due to milk entering the airways during sucking [106]. Those with intestinal fragment atrophy are unable to pass meconium and feces, which initially leads to painful constipation and over time death [107]. For some anatomical malformations, surgical correction may be considered. For example cleft palate surgery [106,108] and surgical operation of the posterior gastrointestinal obstruction [107,109] have been reported with variable success. However, in most cases and with significant severity of defects, euthanasia of affected puppies is often chosen to avoid unnecessary suffering. 

The diagnosis of malformations is often difficult and many of them remain undetected mainly due to a lack of clinical examination or inaccurate clinical examination or the initial absence of clinical signs. Investigation of malformations in newborns immediately after birth is fundamental because an early diagnosis of these conditions could lead to timely clinical interventions and help to minimize mortality.

### 4.2. Birth Weight

Birth weight that reflects intrauterine growth is one of the most important determinants of neonate survival [103,110]. Puppies weighing 25% less than the average weight of a newborn in the given breed are suspected to have a significantly higher mortality rate [111]. The risk of death for puppies characterized by low birth weight is 12 times higher compared to other newborns of normal birth weight from the same litter [112]. Low birth weight is usually followed by fetal immaturity that limits the adaptation of the newborn to the postnatal environment. Such puppies in comparison to average neonates of the same litter have a lower ability to maintain proper body temperature due to a higher body surface/weight ratio and are prone to hypothermia [113]. Moreover, puppies with low birth weight have reduced energy supplies, are less vigorous, and as a result, cannot compete with the rest of the puppies in suckling and thus do not take a sufficient amount of colostrum, which makes them prone to hypoglycemia and dehydration [103,111]. As a result, such newborns would be susceptible to developing growth and neurocognitive deficiencies, cerebral palsy, intracranial hemorrhage, sepsis, hyaline membrane disease, apnea, and retrolental fibroplasia [114].

Factors that influence birth weight could be classified as maternal, fetal, and placental [115]. Dam size, weight, body condition, age and breed, environmental conditions, as well as litter size were described as important variables that affect fetal weight [103,116]. Due to the fact that birth weight reflects intrauterine nutrition of the fetus, the condition of the placenta is one of the basic factors affecting the newborn [113]. Numerous studies have been carried out on human and other animal species that provide information on the relation between the birth weight and placenta [117,118,119,120,121]. Several studies were conducted on dogs [113,122]. In small and toy breeds, authors revealed a positive correlation between the birth weight of a puppy and the placental weight and its total area [113]. The total area of the placenta, transfer zone area, and total vascular area were also proven to correlate strongly with the placental weight [113]. The research focused on the histological examination of the placenta revealed that necrosis was a frequent finding in dogs. However, it was shown that only multifocal-confluent necrosis was associated with a higher risk of newborn death [122]. The placental examination should always be considered as it may provide prognostic information about the puppy’s future development. However, further investigation on a larger scale on breeds from the remaining groups should still be carried out.

### 4.3. Noninfectious Conditions in Newborns

All newborns deserve great attention at this critical moment shortly after birth. The birth weight and daily gains should be carefully recorded during the first weeks of life because adequate growth reflects the puppy’s vitality, health, and proper development [103,111]. Stasis in weight gain is often the first alarming signal in the case of most neonatal diseases. It indicates that the newborn is, for some reason, weakened and does not take enough food [92]. 

The body of a newborn dog works very differently from an adult dog’s body. It is worth noting that in almost every case, the particular disorders of homeostasis in newborns occur together and their symptoms overlap. 

#### 4.3.1. Hypoxia

Hypoxia is the first emerging state responsible for 60% of all neonatal deaths. The oxygen deficits in neonates usually start from the dysfunction of umbilical circulation that can be caused by prolonged and/or complicated parturition, i.e., umbilical cord vessel compression or rupture, as well as too early placental detachment while a puppy is still in the birth canal [92]. In addition, it was reported that puppies born in posterior presentation were more susceptible to respiratory and metabolic acidosis than those born in anterior presentation [123]. Furthermore, administration of oxytocin, anesthetic agents, surgical preparation, and the cesarean section may also contribute to newborn hypoxia [92]. Mildly hypoxic neonates are able to shift circulating blood from intestines, kidneys, spleen, or skin and towards heart, brain, diaphragm, and adrenal glands. Severe oxygen deprivation decreases fetal heartbeat, leading to tissue hypoxia and ischemia and finally multiorgan failure. Affected puppies are more prone to amniotic fluid aspiration, and their mucous membranes become less resistant to the pathogens’ penetration [92].

#### 4.3.2. Hypothermia

The next noninfectious condition that may threaten the life of the newborn is hypothermia. Immediately after birth, any neonate is introduced into the adversely cool environment in comparison to the intrauterine conditions [111,124]. The cooling sensation is escalated by the amniotic fluid residuals. Hypothermia could lead to a significant drop in the heart rate (200–250 bpm at 35.6 °C vs. 40–50 bpm at 21.1 °C), respiratory rate decrease, and loss of suckling reflex, which in turn can cause dehydration and gastrointestinal disorders. Such puppies would be much more susceptible to infections (e.g. herpes virus, bacteria, and opportunistic pathogens) [92]. As thermoregulation is not fully developed in newborn puppies and they are not able to shiver until day 7 of life, to compensate for the temperature loss, they can only use the high energy-consuming thermal conduction [111]. Therefore, the body temperature of a puppy strictly depends on the efficiency of the mother’s care. If the dam’s maternal instincts are inadequate, the proper room temperature and additional heat sources are essential for puppies’ survival [111]. 

#### 4.3.3. Hypoglycemia

Hypoglycemia might be the consequence of the low core temperature or occur separately with the suckling failure due to a variety of reasons. A neonate is born with immature liver and the energy stored as hepatic glycogen is usually enough only for the first day of life [92,125]. Moreover, some factors such as mother’s malnutrition or insufficient nutrition during pregnancy could reduce the newborn’s glycogen supplies. Within 8–12 h after being born, a puppy is forced to rely on colostrum/milk intake to maintain the proper glucose blood level. Reduced or no food intake would result in rapid glycogen reserves depletion and the development of hypoglycemia with typical symptoms of nervousness, vocalization, irritability, and intense hunger that, if not corrected, would be followed by lethargy, mental dullness, depression or stupor, seizures, tremors, and finally the death of the puppy [92]. It is worth noticing that the severity of hypoglycemia symptoms does not always correspond to relatively low blood glucose levels. Moreover, the clinical condition reflects the puppy’s energy reserve. That explains why empirically based treatment with intravenous or oral or glucose in many cases does not lead to a clinical improvement in the neonate [123].

#### 4.3.4. Dehydration

Dehydration represents another homeostatic disorder which is usually a result of a non-properly functioning excretory system, but can also occur with inadequate milk intake. Newborn’s kidneys are not fully developed at birth and need a minimum 2–3 weeks to undergo nephrogenesis to become fully functional [111,125]. The early kidney filtration is characterized by a slow clearance of fluids, increased sodium loss, and, most importantly, the inability to conserve water. That is why neonates are extremely susceptible to dehydration [111]. It is important to remember that water turnover rate in pups is double than in adult dogs and they demand an intake of approximately 132–220 mL/kg/day [111]. Usually, a sick puppy, independently of cause, brought to a veterinary clinic presents a set of common symptoms such as low body temperature, malnutrition, and dehydration [111].

### 4.4. Neonatal Diarrhea

Neonatal diarrhea most commonly results from improper postnatal care and nutrition; however, it can also be caused by infectious agents, especially in the case of high pathogen exposure or adverse environmental conditions. The most common cause of non-infectious diarrhea is overfeeding the puppy or offering unsuitable food [92,126]. Most often it is noted in orphaned puppies and underfed neonates, which require complementary nutrition [92,126]. Thus, it is essential to choose a well-composed milk replacer and carefully calculate the intake for the puppy weight, according to the following rule: 20% of actual body weight per 24 h divided into 6–8 portions [92]. Poorly balanced diet, wrong feeding schedule, or inaccurate amounts would contribute to illnesses and other health problems.

### 4.5. Passive Immune Transfer and Colostrum Intake

It has to be remembered that the primary reason for the majority of postnatal disorders is the insufficient consumption of colostrum, and thus ineffective passive immunity transfer [14,127,128]. Only 10–20% of the mother’s IgG goes through the endotheliochorial placenta in dogs. However, other authors report that puppy serum IgG concentration before colostrum intake contains only 5% of immunoglobulins G compared to adult dogs [129]. The colostrum intake immediately after birth is essential for neonatal survival, development, and further health [130]. Colostrum, apart from IgG, IgA, and IgM, essential for passive immunity of a newborn, also contains nutrients, lysozymes, lactoferrins, white blood cells, cytokines, hormones (cortisol, insulin, thyroxin, somatotropin, growth hormone), specific microbiota, and several growth factors (insulin-like growth factors, epidermal growth factor, nerve growth factor), all needed for proper development [61,129,130]. The most important factor in colostrum intake is the time of ingestion which should not be later than 12–16 h of life [61,130]. This limitation is an effect of neonate digestive wall differentiation. From the moment of birth up to 4 h of life, around 40% of colostrum immunoglobulin (Ig) could be absorbed. This ability decreases gradually up to 12–16 h after parturition, when the junctions between enterocytes become tight and the intestinal wall becomes impermeable for Ig and thus its absorption is not possible any longer [130]. Since the sufficient intake of colostrum controls the risk of newborn puppies’ mortality, colostrum deprivation greatly increases the risk of necrotizing enteric disease and septicemia [130]. 

In the case of the lack of colostrum or its insufficient supply, puppies need to be hand-reared using commercial or homemade milk replacer. However, balancing the formula to cover the needs for nutrients, energy, and volume might be quite challenging, especially for the homemade one. Breeders can also look for a foster dam with a litter of similar age to ensure sufficient immune protection [130]. It was also reported that the oral administration of canine serum immediately after birth resulted in sufficient protective IgG level (2.3 g/L) in puppies’ serum [131]. It is recommended that plasma donors live in the same environment. Moreover, promising results were obtained with the use of serum containing specific antibodies against common canine pathogens [131]. Many products are available on the market; unfortunately, no milk substitute can completely replace the colostrum or natural milk. Immunoglobulins present in some of the formulae can, to some extent, replace the effect of colostrum by coating the intestinal epithelium and creating a barrier against pathogens’ adhesion and translocation to the bloodstream [131]. Some authors also suggested the establishment of dog colostrum banks, similar to farm animals’ husbandry [131]. 

#### Toxic Milk Syndrome

Another non-infectious risk factor which could appear in newborns is toxic milk syndrome (TMS), caused by the milk being contaminated with bacterial toxins [132]. The most common cause of TMS is acute mastitis or metritis present in dams [132]. Toxic milk syndrome usually affects puppies from birth to two weeks of age. The pups become weak and cry intensively. In most cases, diarrhea and intestinal gas accumulation occur. Frequently, due to intensive diarrhea, the puppy’s anus becomes red and swollen. In the authors’ own observations, often the strongest and the most eager to suckle puppies are affected first. If TMS is suspected, suckling should be immediately discontinued, puppies should be fed with milk replacer, and suitable treatment in a dam should be started.

### 4.6. Maternal Care

Dam’s behavior after parturition is strictly related to the offspring’s survival, and the lack of effective maternal care exposes pups to environmental factors dangerous to their health [133,134]. The most common signs of maternal neglect are behavioral: the refusal to permit nursing or abandoning the litter; and physiological: the complete lack of lactation or insufficient lactation. For that reason, the most common effect of maternal neglect is initially hypothermia followed by hypoglycemia and dehydration [92]. On the other hand, some bitches may present excessive maternal behavior characterized by very intense licking and cleaning, which could also lead to hypothermia of a newborn [133]. Breeders can be assured that in abandoned puppies, accurate environmental conditions, scrupulous nurture, and suitable diet should be sufficient to raise a healthy litter. It should also be remembered that maternal care is essential not only for puppies’ health, but also for their socialization and behavioral development [133]. 

The most important conclusion regarding postnatal factors interfering with puppy survival is that most of those factors could be avoided with proper and conscious postnatal care. That is why understanding the mechanism of neonatal problems is essential for successful breeding [103].

## 5. Infectious Factors

A physiologically immature puppy without proper neonatal care will be more prone to suffer from inadequate colostrum intake, and thus will have deficient immunity and increased risk of infection. A bitch may come into contact with different microorganisms at different stages of life. The outcome of pregnancy and the viability of the offspring are most influenced by dams’ immunization (vaccinations), mating hygiene, care during pregnancy and perinatal period, as well as the conditions in which the mother and her puppies are housed for the first few weeks of their lives. Infectious agents are considered the second main cause of a high mortality rate after dystocia [92]. Microorganisms can be transmitted from bitch to pups during pregnancy through the placenta, during delivery, and later from vaginal and oronasal discharges, feces, urine, or milk [135].

### 5.1. Bacterial Agents

Bacterial agents most commonly found in pregnancy loss and increased neonatal death include *Escherichia coli*, *Staphylococcus* spp. *and Streptococcus* spp., *and Klebsiella pneumoniae* [92,135,136,137]. Those detected less frequently include bacteria such as *Brucella canis*, *Proteus mirabilis*, *Pseudomonas aeruginosa*, *Mycoplasma* spp., and *Campylobacter jejuni* [138,139,140,141,142]. Their presence can be associated with abortion, stillbirths, reduced neonatal viability, and even temporary or permanent infertility in adult animals [135]. In the course of most bacterial infections, septicemia is considered the leading cause of death in puppies under 21 days of age [135]. The course of the disease is usually hyperacute and the death occurs shortly thereafter. However, subacute cases have also been observed [4]. The care of a newborn suffering from septicemia is very difficult due to the very vague clinical signs and the rapid progress of the disease, which significantly hinders diagnostic and therapeutic efforts; hence, the prognosis in such cases is very cautious [92]. Among many indicators reviewed for their usefulness in the assessment of mortality risk, the viability assessment carried out using the modified APGAR scale and the colostrum intake measured by blood glucose concentration during the first 24 h of life has proven to be useful for canine neonates [4,94]. It should also be mentioned that ticks can be a source of infection with dangerous bacteria. Cases of fetuses being infected by their mother suffering from tick-borne diseases caused by the *Anaplasma platys* bacterium have been documented [143,144]. The detection of bacterial DNA in newborn pups of seropositive dams in the absence of clinical signs is suggestive of the ability of the bacterium to cross the placental barrier. Moreover, its capability to infect fetuses as early as the first half of pregnancy has also been demonstrated [143]. More research is needed to determine the effects of infection during the fetal period on the life and health of the offspring, as well as to raise awareness among vets and owners to administer appropriate bacterial prophylaxis to their pets.

### 5.2. Viral Agents

Viruses are a common cause of reproductive failure in companion animals. Their small size facilitates crossing the placental barrier, leading to pregnancy losses either by transplacental transmission itself and direct infection of embryos or fetuses or, less frequently, by severe debilitation of pregnant animals in the absence of congenital infection [145]. Canine parvovirus (CPV-1) and canine herpesvirus (CaHV-1), among others, are mainly responsible for such problems. In the case of herpesvirus infection, litter size disproportion often develops, neonates are born dead or weak, and mortality in the first 8 weeks of life is usually high [146]. In the case of canine parvovirus infection, depending on the stage of pregnancy in which the infection occurred, embryonic death and resorption are possible in the early stages, while stillborn or weak pups are observed in the later stages. Acute infection in puppies in the first days of life is present with vague general signs (no food intake, hypoglycemia, fever) and usually soon leads to the puppy’s death [147]. The morbidity of the virus is usually 100%, and mortality among puppies younger than 6 months without medical treatment reaches 91%, which can be reduced with suitable treatment [148]. The effective management of viral infections in breeding dogs is primarily based on extensive preventive measures. Various immunization protocols are available, including vaccination against canine parvovirus [149] and canine herpesvirus [146]. Vaccination is the most effective way to control the spread of both infections in dogs and to prevent the development of clinical forms of diseases. Vaccination eliminates the risk of infection during and after pregnancy, increasing the pup’s chances of survival.

### 5.3. Parasitic Agents

When talking about infectious factors, one cannot forget the problem associated with infections with intracellular parasites such as *Toxoplasma gondii* and *Neospora caninum*. Toxoplasmosis in dogs is much less common than in cats. Most often it leads to immunosuppression and the onset of neurological symptoms: seizures, nerve deficits, ataxia, or paralysis can be evident [150]. Infection-prone pregnant bitches are a serious concern. Parasitemia can cause placentitis, followed by the spread of tachyzoites to the fetus, and can cause miscarriage. Fetal resorptions and sudden infant death have also been reported [151]. *T. gondii* has also been isolated from pups of seropositive dams without clinical signs [150]. Toxoplasmosis is frequently associated with secondary infections in dogs. When combined with viral infections, such as the distemper virus (CDV), it can cause the death of the entire litter due to a complete immune failure in very young animals (up to 30 days of age) [152]. The second intracellular parasite is the protozoan *Neospora caninum* [153]. Dogs can be intermediate or definitive hosts, while infection occurs mainly through contact with contaminated water or food containing cysts. The horizontal transmission of the parasite from mother to fetus through the placenta is also possible [153]. Robbe et al. reported that pregnancy might be a predisposing factor for the *Neospora caninum* infection, which usually results in abortion or the birth of weak puppies that die shortly after [154]. In Australia, a case of a bulldog litter born from a seropositive mother was reported, in which one of the seven pups died from a multisystemic infection caused by *Neospora caninum* [155]. The pup was the smallest of the litter and was reported to have signs of weakness, lack of sucking reflex, and difficulty breathing. When necropsy was performed, diffuse pulmonary edema, inflammatory changes in internal organs, and acute myocarditis were detected. Studies conducted in Italy [154], Iran [156], Brazil [157], and Australia [158] indicated that parasitic diseases in breeding dogs are underestimated and often overlooked despite the significant prevalence of toxoplasmosis and neosporosis in the companion dog population. It should be noted that they are related to the trend of feeding raw animal meat in the BARF diet, which, at least for this reason, should not be recommended for feeding pregnant females, as it can be a potential source of infection with these protozoa [159,160].

## 6. Conclusions

The neonatal period is a challenging time of adaptation for any puppy to life outside their mother’s body. In our review, we have discussed a variety of factors that affect puppy viability in the early stages of life. The order of discussion was guided by the natural course of pregnancy, birth, and the neonatal period. The viability of puppies depends first on the health of the mother and the environment that affects her in different ways, and then on the maturity of the adaptive mechanisms developed by those puppies during embryonic and fetal development.

For the early detection of the first signs of any emerging abnormality, the fundamental factor is conducting examinations and observing the pregnant bitch. Regular ultrasound and electrocardiographic monitoring enable rapid diagnosis of abnormalities, which, in turn, increases the chance of effective implementation of therapeutic measures [5,78,79,80,81,82,84]. This is especially true in the case of potential perinatal complications, because the impact of delivery on pup viability should always be kept in mind. The emergency cesarean section carries far greater risks than natural birth or planned surgery, and delaying the decision can often be fatal to the viability of the pups [5]. Therefore, the safest solution would be a planned cesarean section in the case of bitches known to be prone to perinatal complications, either due to breed predisposition or previous history. After the surgery, it is necessary to perform a quick evaluation of newborns, usually using the modified APGAR score [94] and the NVR [5], which are highly effective in identifying weak pups that require special care.

The cesarean section is also a challenging procedure due to the adequate intra- and postoperative care. For the safety of milk-sucking puppies, medications given to the mother are often highly restricted. However, after all, they are essential for dams’ welfare and could significantly reflect the quality of care a female provides for her puppies [100,133,134]. In the authors’ opinion, the problem of providing adequate analgesia protection to the bitch after a cesarean section is urgent and certainly requires further research to improve current surgical protocols. However, it should be remembered that once a puppy does not take milk, especially not colostrum, the risk of developing immunity problems would increase. Any factors that weaken a newborn’s immunity drastically increase its susceptibility to infections. Thus, it is crucial to provide diligent care and hygiene for the bitch and her offspring. The risk of certain infections can be reduced through regular vaccinations. 

In conclusion, all collected information focuses on factors influencing the viability of puppies during their most critical infancy period. The authors hope that this review would be helpful both for scientists and practitioners to grasp the picture of the vastness of the interrelationships during the perinatal period that determine neonatal health and welfare. It should also be mentioned that canine reproduction currently focuses on pedigree dogs only. Hence, inbreeding and focusing solely on exterior characteristics significantly increased susceptibility to perinatal complications [10]. This leads to a situation observed more and more often nowadays, where reproduction in some breeds, mainly brachycephalic, is impossible due to their anatomy and physiology, without medical assistance performed by veterinarians [21]. The ethics of such a practice is difficult to assess due to divergent opinions among scientists, physicians, and breeders involved in small animal reproduction [17,161]. Cooperation between science and veterinary medicine can contribute to a more effective accumulation of knowledge in the field of canine neonatology and thus a further improvement in the quality of services provided. 

## Data Availability

Not applicable.
